# Highly proliferative neuroendocrine carcinoma – influence of radiotherapy fractionation on tumor response

**DOI:** 10.1186/1748-717X-3-13

**Published:** 2008-05-19

**Authors:** Anne Hansen Ree

**Affiliations:** 1 Division of Cancer Medicine and Radiotherapy, The Norwegian Radium Hospital, Rikshospitalet University Hospital, 0310 Oslo, Norway; 2 Faculty Division Akershus University Hospital, University of Oslo, 0318 Oslo, Norway

## Abstract

A 45-year-old white male presented to our department with postoperative recurrence of gastrointestinal poorly differentiated neuroendocrine carcinoma manifesting as lymph node dissemination and a solitary implantation metastasis in the rectovesical pouch. Following disease progression on chemotherapy, the patient was treated with radiotherapy using either a conventional daily treatment or an accelerated hyperfractionated protocol to separate sites of disease progression. Using serial CT scan assessment, changes in cross-sectional area of the separately treated metastatic lesions were evaluated for determination of therapy response. The accelerated hyperfractionated radiotherapy appeared to limit the rate of tumor growth to a greater degree than the conventional fractionation schedule. Of uttermost importance, in this palliative setting, the patient completed the intensified radiotherapy regimens with acceptable acute toxicity. Given the proliferative capacity of poorly differentiated neuroendocrine carcinomas of the gastrointestinal tract, radiotherapy may be a therapeutic supplement to chemotherapy, which represents the main treatment option in this tumor entity. Importantly, tumors with a capacity for rapid proliferation and regeneration may be particularly sensitive to the use of intensified fractionation protocols in clinical radiotherapy.

## Findings

Radiotherapy is typically administered by fractionated schedules to allow normal tissue recovery from sublethal damage between each treatment fraction. However, surviving tumor cells also proliferate during the interfraction periods, and in addition, accelerated repopulation is a recognized contributor to treatment failure. The rate of repopulation often increases with time during radiotherapy treatment, and accelerated fractionation protocols have therefore been suggested as potentially advantageous by shortening the overall treatment time [1]. Moreover, hyperfractionated accelerated schedules, using a lower dose per fraction combined with an increased number of fractions and shortened overall treatment time, may also improve tumor control. The radiobiological principles of altered fractionation schedules, treatment time, and total dose have been most extensively tested in clinical trials on head-and-neck squamous cell carcinoma [2,3].

Undifferentiated small-cell carcinoma of the gastrointestinal tract represents a rare tumor entity and may originate in any site within the gastrointestinal tract. Most patients either present with or rapidly develop disseminated disease. Traditionally, surgery has been a significant treatment option in locoregionally limited disease, although many patients ultimately have systemic relapse. These tumors are generally highly sensitive to chemotherapy, but in extensive disease, responses to chemotherapy are usually of short duration, and progression after first-line therapy is often aggressive [4]. Available data on clinical situations for which radiotherapy may be of benefit, is principally as part of the management of patients with limited-stage disease of the esophagus, stomach, or rectum [4,5].

A 45-year-old white male, never-smoker, presented to a local hospital with a 1-month history of intermittent bleedings per rectum. His medical background was unremarkable. Lower endoscopy revealed an ulcer in the terminal ileum adjacent to the ileocecal valve, and the patient proceeded to resection of the terminal ileum and right hemicolon. Histological examination (See additional file 1: Histology of the primary tumor surgical specimen) confirmed a transmural small-cell carcinoma containing nests of abnormal tubular and glandular structures, associated with vessel invasion, abundant in mitotic figures and with high Ki67 proliferation index (90%), and showing immunohistochemical positivity for synaptophysin and chromogranin and lymph node involvement, consistent with a poorly differentiated neuroendocrine carcinoma of the gastrointestinal tract [6,7].

**Figure 1:**
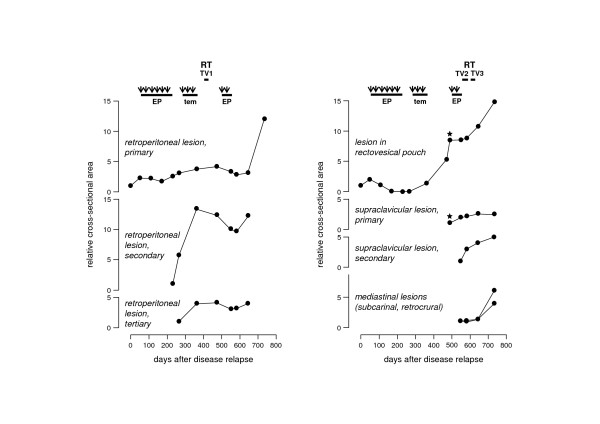
**Poorly differentiated neuroendocrine carcinoma – progression and therapy response of disease manifestations**. Repeated helical CT scannings were accomplished in the course of the disease relapse (when the patient was referred to our institution), which was defined as day 1. On the basis of the diagnostic description of contrast-enhancing lesions in the transverse view, cross-sectional area (the maximum diameter multiplied by perpendicular bisector) of each lesion was calculated, according to the established World Health Organization criteria for evaluation of tumor response to cytotoxic therapy. Filled circles: lesion size at each CT record, determined relative to the cross-sectional area at first appearance (set to the value of 1). Stars: the radiological review at day 492, done by MRI. Thick lines: duration of the therapy regimens. Arrowheads: time for start of chemotherapy cycles. EP: chemotherapy consisting of etoposide and cisplatin. tem: chemotherapy consisting of temozolomide. RT: radiotherapy. TV1: target volume 1 (pathologic retroperitoneal lymph nodes). TV2: target volume 2 (macroscopic pelvic tumor). TV3: target volume 3 (pathologic supraclavicular lymph nodes).

The patient was referred to our department four months after the abdominal surgery when abdominal-pelvic CT scanning had revealed an enlarged, retroperitoneal lymph node (16 mm × 18 mm in the transverse view) located between the abdominal aorta and inferior vena cava and a lesion with peripheral contrast enhancement and decreased central density (19 mm × 32 mm in the transverse view) located in the rectovesical pouch. Because of waiting time until admittance, a new CT scan was performed (51 days after the previous, which was defined as the day of disease relapse), confirming an additional ≥ 2-fold increases of cross-sectional lesion area (Figure 1). The patient proceeded to ultrasound-guided biopsy confirmation of the retroperitoneal nodal relapse prior to commencing chemotherapy consisting of etoposide (100 mg/m^2 ^daily, on days 1–3) and cisplatin (45 mg/m^2 ^daily, on days 2–3) in 4-week cycles (Figure 1; EP: etoposide/cisplatin). CT evaluation of tumor response was initially performed after every two treatment cycles. Both pathologic lesions showed significant initial responses; however, disease progression was noted at the retroperitoneal region after the sixth cycle (Figure 1), resulting in discontinuation of the etoposide/cisplatin chemotherapy. Following additional CT evidence of disease progression (266 days after the diagnosis of disease relapse), the patient proceeded to second-line temozolomide chemotherapy, according to clinical experience from Uppsala University Hospital in Sweden [8]; however, both the retroperitoneal and the pelvic lesions progressed on this treatment (Figure 1; tem: temozolomide).

Given the insensitivity to chemotherapy, the patient was assessed for palliative radiotherapy to facilitate pain control at the retroperitoneal site (See additional file 2: Abdominal CT scan 364 days after the diagnosis of disease relapse). To take account of the high proliferation index noted in the initial and repeat biopsy specimens, a hyperfractionated accelerated protocol was considered to be of therapeutic benefit. Provided a tolerance of 50 Gy with conventional fractionation (2 Gy daily) for the spinal cord and intestines, if one-third of the small bowel is exposed, and an α/β ratio of 3 for these tissues, 37 fractions of 1.5 Gy might be tolerated. Hence, the patients proceeded to a treatment schedule of 48 Gy delivered in 16 treatment days with twice daily fractionation, using anterior-posterior radiation fields (Figure 1; RT TV1: radiotherapy, target volume 1). An abdominal-pelvic CT scan at two months after completion of the radiotherapy (474 days after the diagnosis of disease relapse) showed disease stabilization at the retroperitoneal site (Figure 1).

Following the retroperitoneal radiation course, the patient experienced voiding difficulties, consistent with pelvic disease progression with tumor extension to the seminal vesicles and the anterior rectal wall shown on the CT scan, and additional MRI confirmed tumor infiltration at the prostatic urethral lobe and posterior bladder wall (See additional file 3: Pelvic MRI examination 492 days after the diagnosis of disease relapse). Additional disease progression was noted in the left supraclavicular fossa. A trial of repeat etoposide/cisplatin chemotherapy was commenced; however, disease progression was noted and the disease was deemed to be refractory to chemotherapy (Figure 1; EP: etoposide/cisplatin).

Further radiotherapy to the two sites of progressive disease was considered with the goals of symptom control and disease constraints. The macroscopic pelvic tumor was treated using a conventional fractionation protocol to a total dose of 50 Gy (2 Gy in daily fractions) using anterior-posterior radiation fields (Figure 1; RT TV2: radiotherapy, target volume 2). However, at treatment completion, the patient could no longer control micturition or defecation, which was clinically interpreted as pelvic disease progression. The supraclavicular lymph node metastases were subsequently treated using a CT-planned approach to 51 Gy using a hyperfractionated accelerated protocol (1.5 Gy twice daily fractionation in 17 treatment days) (Figure 1; RT TV3: radiotherapy, target volume 3). Towards the end of this treatment, the patient experienced Common Toxicity Criteria Grade 1 pharyngeal toxicity. Repeat CT examination (646 days after the diagnosis of disease relapse) confirmed disease progression at the pelvic site, whereas a mixed response, in part with disease stabilization, was observed at the supraclavicular site (Figure 1). This pattern of differential response was documented until the time of patient death, two years and seven months after the primary surgery.

Given that nodal tumor masses are regular ovoid structures that show proportional changes in all dimensions, measurements of bidimensional tumor size (cross-sectional area) are fully in accordance with volumetric measurements when assessing treatment response [9]. Moreover, when almost all tumor cells are actively dividing, as in this patient, it is conceivable that any change in tumor size after a given treatment may be a direct consequence of the clonogenic regeneration rate, and it may therefore be possible to compare the efficacy of different radiotherapy fractionation regimens. These considerations are contingent on radiologic evaluation by a single imaging modality. In this patient, MRI was done once (to visualize intrapelvine structures; for practical reasons, cervical MRI was done simultaneously). Compared with CT scans, the MRI examination probably overestimated tumor sizes.

In extrapulmonary small-cell carcinomas, no randomized trials are reported to guide patient management, and few data exists on patient outcome after radiotherapy. Given the rarity of these tumor entities, treatment approaches have typically been extrapolated from trials designed for limited-stage small-cell lung carcinoma [10,11]. As outlined in a recent review, radiotherapy for small-cell carcinoma of the gastrointestinal tract has been prescribed in a wide variety of doses, ranging between 30 and 66 Gy, the higher doses typically administered with chemotherapy and with curative intent [4]. In a recent retrospective, single-center study on the use of radiotherapy in limited-stage extrapulmonary small-cell carcinoma, only 2 of 18 reported patients had been treated with hyperfractionated protocols [12]. The clinical observations in this patient and a previously reported case [6] suggest a role for radiation also with clearly palliative indications in extensive gastrointestinal tract small-cell carcinoma.

In this patient, the independent courses of accelerated hyperfractionated radiotherapy to the retroperitoneal and supraclavicular nodal sites appeared to result in a greater degree of tumor control when compared to the conventional daily fractionation course delivered to the pelvic recurrence. The 2-Gy Biological Equivalent Dose delivered by the intensified fractionation schedules equals 44–48 Gy, and the patient was able to complete both of the intensified radiotherapy regimens with acceptable acute toxicity. This aspect, together with the advantage of a reduction in overall treatment time, are critically important objectives of optimized radiation oncology care in the palliative therapy setting.

## Competing interests

The author declares that she has no competing interests.

## Authors' contributions

AHR conceived of the patient's treatment and management, supervised chemotherapy administration and radiotherapy treatment planning and accomplishment, performed interpretation and clinical approval of the radiologic assessments throughout the treatment course and for this presentation, and conceived, prepared, and approved the manuscript.

## Supplementary Material

Additional file 1 **Histology of the primary tumor surgical specimen**. The figure is displayed in PDF format. The panels represent sections with hematoxylin and eosin staining (left column) or immunohistochemical staining for Ki67 (right column) and depict an ileal, transmural small-cell carcinoma containing nests of abnormal tubular and glandular structures and with a high fraction (90%) of Ki67-positive tumor cells. Click here for file

Additional file 2 **Abdominal CT scan 364 days after the diagnosis of disease relapse**. The figure is displayed in PDF format. The images were generated by multislice CT technique (with the liver parenchyma in contrast-enhanced portovenous phase), and the two representative images in transverse view (left) and reconstructed image in coronal view (right) display retroperitoneal lymph node metastases (M) located between the abdominal aorta and inferior vena cava. The round structure located adjacent to the left kidney was a parapelvine cyst, which remained unaltered throughout the disease course. Click here for file

Additional file 3 **Pelvic MRI examination 492 days after the diagnosis of disease relapse**. The figure is displayed in PDF format. The two representative MR images are oblique and T_2_-weighted and display the tumor (periphery indicated by arrows) located in the rectovesical pouch, infiltrating into the posterior wall of the bladder (B) and the urethral lobe of the prostate (P). Tumor extension to the anterior rectal wall was observed on an accompanying CT scan. Click here for file

## References

[B1] Kim JJ, Tannock IF (2005). Repopulation of cancer cells during therapy: an important cause of treatment failure. Nat Rev Cancer.

[B2] Bourhis J, Overgaard J, Audry H, Ang KK, Saunders M, Bernier J, Horiot JC, Le Maître A, Pajak TF, Poulsen MG, O'Sullivan B, Dobrowsky W, Hliniak A, Skladowski K, Hay JH, Pinto LH, Fallai C, Fu KK, Sylvester R, Pignon JP (2006). Hyperfractionated or accelerated radiotherapy in head and neck cancer: a meta-analysis. Lancet.

[B3] Budach W, Hehr T, Budach V, Belka C, Dietz K (2006). A meta-analysis of hyperfractionated and accelerated radiotherapy and combined chemotherapy and radiotherapy regimens in unresected locally advanced squamous cell carcinoma of the head and neck. BMC Cancer.

[B4] Brenner B, Tang LH, Klimstra DS, Kelsen DP (2004). Small-cell carcinomas of the gastrointestinal tract: a review. J Clin Oncol.

[B5] Brenner B, Shah MA, Gonen M, Klimstra DS, Shia J, Kelsen DP (2004). Small-cell carcinoma of the gastrointestinal tracts: a retrospective study of 64 cases. Br J Cancer.

[B6] Ree AH (2006). A complex case of rectal neuroendocrine carcinoma with terminal delirium. Nat Clin Pract Gastroenterol Hepatol.

[B7] Klöppel G, Perren A, Heitz PU (2004). The gastroenteropancreatic neuroendocrine cell system and its tumors. Ann NY Acad Sci.

[B8] Ekeblad S, Sundin A, Janson ET, Welin S, Granberg D, Kindmark H, Dunder K, Kozlovacki G, Orlefos H, Sigurd M, Oberg K, Eriksson B, Skogseid B (2007). Temozolomide as monotherapy is effective in treatment of advanced malignant neuroendocrine tumors. Clin Cancer Res.

[B9] Sohaib SA, Turner B, Hanson JA, Farquharson M, Oliver RT, Reznek RH (2000). CT assessment of tumour response to treatment: comparison of linear, cross-sectional and volumetric measures of tumor size. Br J Radiol.

[B10] Schild SE, Bonner JA, Shanahan TG, Brooks BJ, Marks RS, Geyer SM, Hillman SL, Farr GH, Tazelaar HD, Krook JE, Geoffroy FJ, Salim M, Arusell RM, Mailliard JA, Schaefer PL, Jett JR (2004). Long-term results of a phase III trial comparing once-daily radiotherapy with twice-daily radiotherapy in limited-stage small-cell lung cancer. Int J Radiat Oncol Biol Phys.

[B11] Jassem J (2007). The role of radiotherapy in lung cancer: where is the evidence?. Radiother Oncol.

[B12] Soto DE, Eisbruch A (2007). Limited-stage extrapulmonary small cell carcinoma: outcomes after modern chemotherapy and radiotherapy. Cancer J.

