# Supplementary figures and images for: Highly proliferative neuroendocrine carcinoma – influence of radiotherapy fractionation on tumor response
Source: Radiat Oncol. 2008 May 19;3:13. doi: 10.1186/1748-717X-3-13 (PMC2397422; doi:10.1186/1748-717X-3-13)

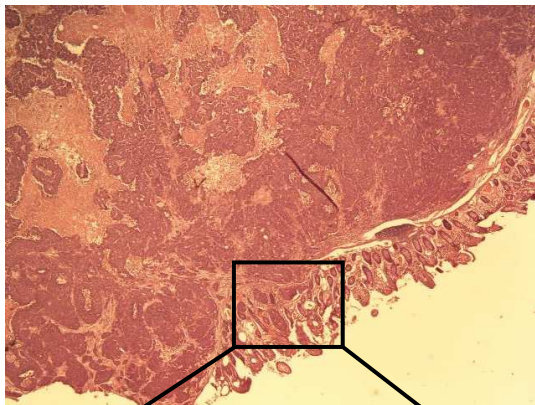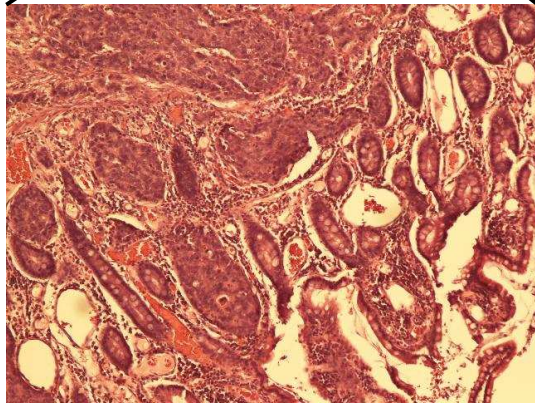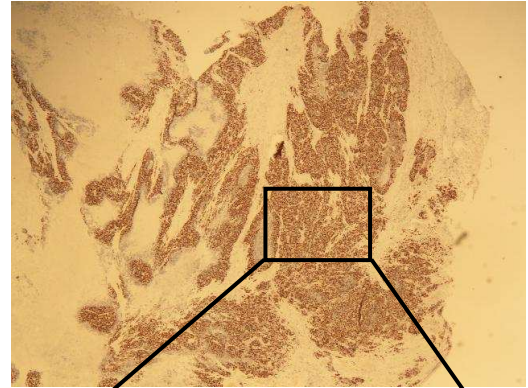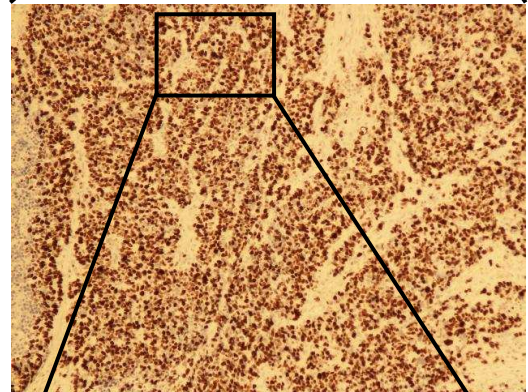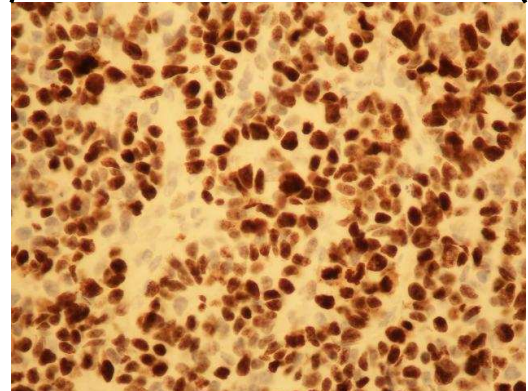

Supplement: Additional file 1 — Histology of the primary tumor surgical specimen. The figure is displayed in PDF format. The panels represent sections with hematoxylin and eosin staining (left column) or immunohistochemical staining for Ki67 (right column) and depict an ileal, transmural small-cell carcinoma containing nests of abnormal tubular and glandular structures and with a high fraction (90%) of Ki67-positive tumor cells. [file 1748-717X-3-13-S1.pdf]

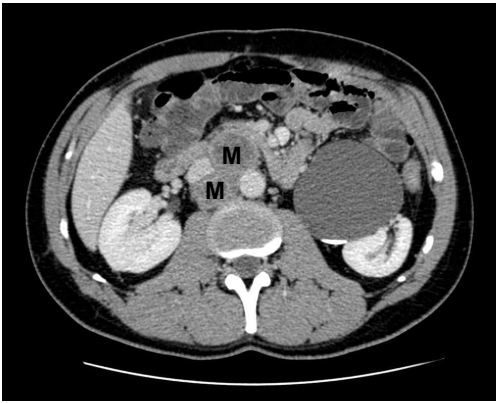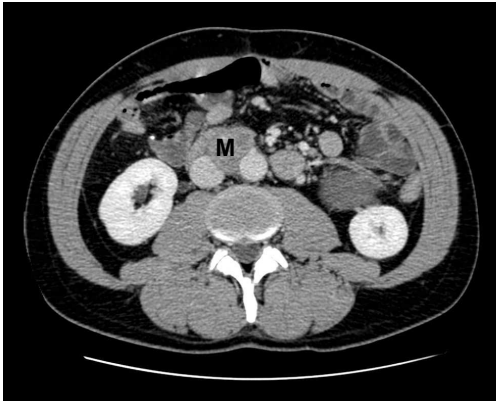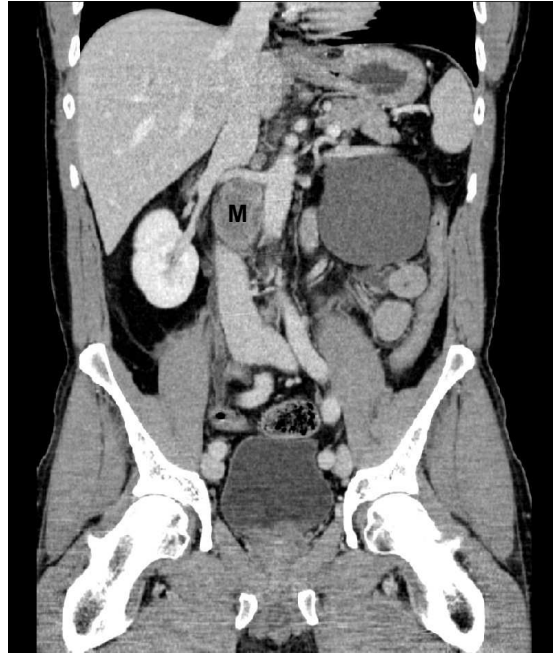

Supplement: Additional file 2 — Abdominal CT scan 364 days after the diagnosis of disease relapse. The figure is displayed in PDF format. The images were generated by multislice CT technique (with the liver parenchyma in contrast-enhanced portovenous phase), and the two representative images in transverse view (left) and reconstructed image in coronal view (right) display retroperitoneal lymph node metastases (M) located between the abdominal aorta and inferior vena cava. The round structure located adjacent to the left kidney was a parapelvine cyst, which remained unaltered throughout the disease course. [file 1748-717X-3-13-S2.pdf]

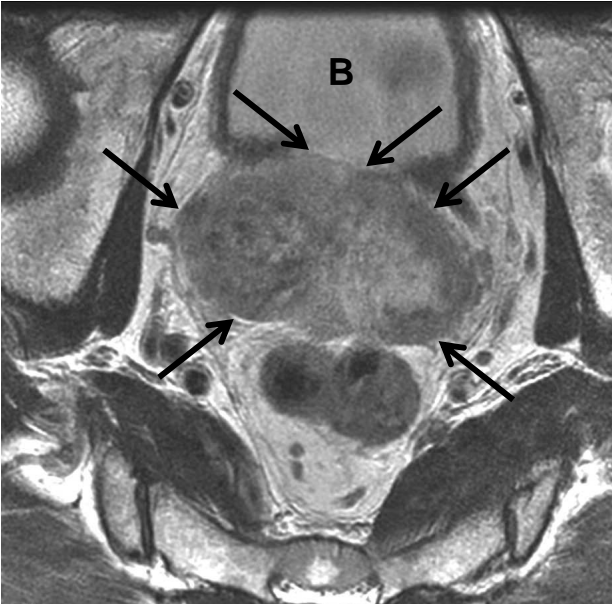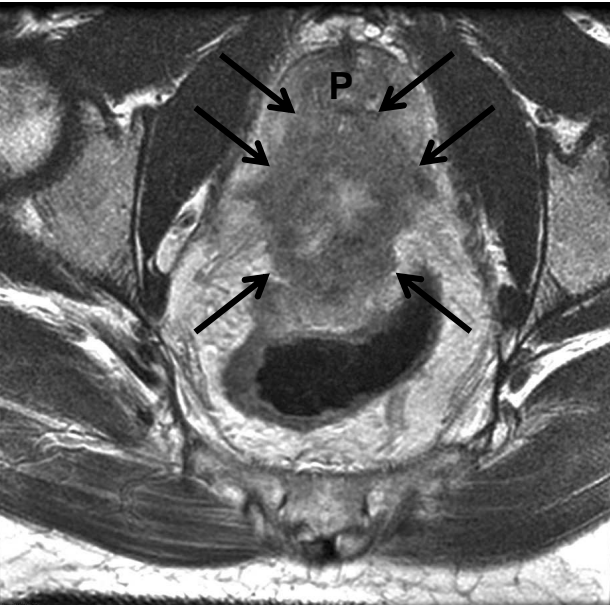

Supplement: Additional file 3 — Pelvic MRI examination 492 days after the diagnosis of disease relapse. The figure is displayed in PDF format. The two representative MR images are oblique and T2-weighted and display the tumor (periphery indicated by arrows) located in the rectovesical pouch, infiltrating into the posterior wall of the bladder (B) and the urethral lobe of the prostate (P). Tumor extension to the anterior rectal wall was observed on an accompanying CT scan. [file 1748-717X-3-13-S3.pdf]
